# Postoperative Trends of ESR and CRP Following Total Hip Arthroplasty in the Indian Population: A Prospective Observational Study

**DOI:** 10.7759/cureus.112289

**Published:** 2026-07-08

**Authors:** Pratyush Sinha, Anil K Juyal, Siraz Malik

**Affiliations:** 1 Department of Orthopaedics, Himalayan Institute of Medical Sciences, Dehradun, IND

**Keywords:** c-reactive protein, erythrocyte sedimentation rate, periprosthetic joint infection, postoperative inflammatory markers, total hip arthroplasty

## Abstract

Background

Total hip arthroplasty (THA) is a widely performed orthopedic procedure used to relieve pain and restore function in patients with advanced hip disorders such as avascular necrosis and osteoarthritis. Following surgery, inflammatory markers such as erythrocyte sedimentation rate (ESR) and C-reactive protein (CRP) commonly increase as part of the normal postoperative inflammatory response. However, persistent elevation may also indicate complications such as periprosthetic joint infection. Understanding the normal postoperative trends of these biomarkers is important for accurate clinical interpretation and early identification of abnormal recovery patterns. The present study aimed to characterize the postoperative temporal trends of ESR and CRP during the first 30 days following uncomplicated THA in adult Indian patients.

Methods

This prospective observational study was conducted in the Department of Orthopaedics at Himalayan Institute of Medical Sciences, Dehradun, over a period of 12 months. A total of 48 adult patients undergoing THA were enrolled using consecutive sampling after obtaining informed consent and ethical approval. Patients with chronic infection, inflammatory diseases, autoimmune disorders, or elevated preoperative ESR and CRP levels were excluded. ESR and CRP levels were measured preoperatively and on postoperative days (PODs) 3, 7, 14, and 30.

Results

The mean age of patients was 43.10 ± 10.49 years, and males constituted 70.8% of the study population. The mean preoperative ESR was 8.94 ± 6.58 mm/hr, which increased significantly to 36.02 ± 5.90 mm/hr on POD 3 and remained elevated on POD 7 before gradually declining to 28.48 ± 5.61 mm/hr on POD 30. A statistically significant variation was observed in ESR levels over time (F = 408.740, p < 0.001). The mean preoperative CRP level was 2.74 ± 1.43 mg/L. CRP showed a marked rise to 131.19 ± 30.36 mg/L on POD 3, followed by a progressive decline to 5.55 ± 3.70 mg/L by POD 30. Changes in CRP levels across postoperative intervals were also statistically significant (F = 499.360, p < 0.001). ESR demonstrated a slower normalization pattern compared to CRP, whereas CRP showed rapid postoperative elevation and faster decline toward baseline values.

Conclusion

ESR and CRP exhibit distinct postoperative kinetics following THA. ESR remains elevated for a longer duration, while CRP rises rapidly and declines more quickly toward baseline levels. Knowledge of these normal postoperative trends may help clinicians differentiate expected postoperative inflammatory responses from abnormal patterns suggestive of infection or other complications.

## Introduction

Total hip arthroplasty (THA) is one of the most successful orthopedic procedures performed to relieve pain and improve mobility in patients suffering from severe hip diseases such as osteoarthritis, rheumatoid arthritis, and avascular necrosis (AVN). In this procedure, the damaged hip joint is replaced with an artificial prosthesis to restore function and improve quality of life [1]. Over the past few decades, the number of THA surgeries has increased steadily because of rising life expectancy, better surgical techniques, and advancements in implant design [2]. More than 400,000 THA procedures are currently performed annually worldwide, and this number is expected to increase further in the coming years [3]. Although THA generally provides excellent outcomes, postoperative complications can still occur and may affect recovery and long-term function.

Among these complications, periprosthetic joint infection (PJI) remains one of the most serious and challenging problems after THA. Even though the incidence of PJI is relatively low, around 1-2%, it accounts for nearly 20% of revision THA procedures. PJI can lead to prolonged hospitalization, repeated surgeries, increased healthcare costs, and poor functional outcomes. The financial burden related to THA-associated PJI is also rising and is projected to reach approximately $753.4 million annually by 2030 [3]. Therefore, early detection and prevention of infection are essential for improving patient outcomes.

To improve the diagnosis of PJI, the Musculoskeletal Infection Society (MSIS) introduced standardized diagnostic criteria in 2011, which were later refined. These criteria include clinical findings, microbiological investigations, histopathology, joint aspiration, and laboratory tests. Among laboratory investigations, inflammatory markers such as erythrocyte sedimentation rate (ESR) and C-reactive protein (CRP) are commonly used as first-line tests because they are inexpensive, widely available, and minimally invasive [4]. These markers are also useful for monitoring postoperative recovery.

ESR is a laboratory test that measures the rate at which red blood cells settle in a tube over time and serves as an indirect indicator of inflammation. Several studies have shown that elevated ESR values after THA may indicate deep infection. Carlsson et al. reported that ESR values above 40 mm/hr or between 30 and 140 mm/hr beyond three months after surgery may suggest septic complications, even in the absence of obvious clinical symptoms [5]. Other studies found significantly higher ESR levels in patients with PJI compared to uncomplicated THA cases, where ESR usually returned to near-normal levels within a few months. Yishake et al. observed that ESR generally peaks during the first postoperative week and gradually normalizes within 3-4 months, whereas persistently elevated values may indicate infection [6]. These findings show the importance of serial ESR monitoring in distinguishing normal postoperative inflammation from PJI.

CRP is another important inflammatory marker commonly used after surgery. It is an acute-phase protein produced mainly by the liver in response to inflammation, tissue injury, or infection. CRP levels rise rapidly after surgery and gradually decrease as healing progresses. Due to its rapid response and short half-life, CRP is considered a sensitive marker for detecting postoperative complications, particularly bacterial infections [7]. Persistently elevated CRP levels or a second rise around postoperative days (PODs) 7-8 may indicate an underlying infection and require further evaluation. Clinical signs such as pain, redness, swelling, and wound discharge are often non-specific in the early postoperative period, making laboratory markers particularly useful in identifying infection [8].

Patients with infected arthroplasties often have significantly elevated CRP and ESR levels. However, both markers may increase after surgery even in the absence of infection because of normal inflammatory responses to surgical trauma. Therefore, many clinicians consider the trend and temporal pattern of ESR and CRP more valuable than isolated readings at a single time point. CRP generally shows a faster rise and decline compared to ESR, making it useful for monitoring early postoperative changes. However, the time required for normalization of ESR and CRP varies among individuals and across studies.

Understanding the normal postoperative trends of ESR and CRP is important for differentiating routine inflammatory responses from early signs of infection. Therefore, the present study aimed to characterize the postoperative temporal trends of ESR and CRP during the first 30 days following uncomplicated THA in adult Indian patients.

## Materials and methods

Study setting, design, and data collection

The present hospital-based observational prospective study was conducted in the Department of Orthopaedics at Himalayan Institute of Medical Sciences, Swami Ram Nagar, Dehradun, over a period of 12 months (January 2025 to December 2025). The present study aimed to characterize the postoperative temporal trends of ESR and CRP during the first 30 days following uncomplicated THA in adult Indian patients. Patients undergoing THA during the study period were recruited after obtaining written informed consent. Ethical clearance for the study was obtained from the Institutional Ethics Committee prior to the commencement of the study (Letter No. SRHU/HIMS/ETHICS/2026/409). Data collection was carried out using a structured case reporting form that included demographic details, clinical findings, laboratory parameters, and postoperative follow-up information.

Sample size and sampling method

The sample size for the present study was determined pragmatically based on feasibility and previous prospective studies evaluating postoperative ESR and CRP trends following THA [9]. As no prior repeated-measures data from our study population were available to perform a formal repeated-measures sample size calculation, a feasibility-based approach was adopted. To improve the reliability of serial postoperative observations and account for potential variability in biomarker measurements, a total of 48 consecutive eligible patients were enrolled during the study period.

Selection of study participants

All patients planned for THA during the study period were screened for eligibility and assessed according to the predefined inclusion and exclusion criteria. Patients undergoing THA were included in the study. Patients with a history of chronic infection, chronic inflammatory diseases, autoimmune disorders such as rheumatoid arthritis, or elevated preoperative (pre-op) inflammatory markers (serum CRP >5 mg/L and/or ESR >30 mm/hr) were excluded from the study. All procedures were performed by experienced orthopedic surgeons following the institution's standard perioperative protocol, including antibiotic prophylaxis, postoperative analgesia, and rehabilitation.

Study procedure

Baseline demographic information such as age and gender was recorded for all patients before surgery. Pre-op ESR and CRP levels were measured in all participants. For laboratory analysis, 2 ml of blood sample was collected separately for ESR and CRP estimation. Quantitative CRP analysis was performed using an immunoassay-based testing kit, with a normal reference range of 0-5 mg/L. ESR estimation was carried out using Wintrobe’s method, with a normal reference range of 0-30 mm/hr. Postoperatively, blood samples were collected on the 3rd, 7th, 14th, and 30th postoperative days for serial measurement of ESR and CRP levels. Patients were also monitored for the development of any postoperative complications during the follow-up period. All blood samples were processed in the same institutional central laboratory using standardized laboratory protocols. Quality control procedures routinely followed by the laboratory were maintained throughout the study.

Data management and statistical analysis

Data entry was performed using Microsoft Excel (Redmond, Washington) spreadsheet software, and statistical analysis was conducted using IBM SPSS for Windows, Version 28.0 (Released 2021; IBM Corp., Armonk, New York). All enrolled patients completed follow-up through postoperative day 30, and no missing outcome data were observed. Continuous variables were expressed as mean ± standard deviation, while categorical variables were presented as frequencies and percentages. The data were tested for normality prior to analysis. Changes in ESR and CRP levels over different postoperative time intervals were analyzed using repeated measures analysis of variance (ANOVA) followed by a Bonferroni post-hoc test for normally distributed data or a Friedman test for non-parametric data. A p-value of less than 0.05 was considered statistically significant.

## Results

The present study included 48 patients undergoing THA, with a mean age of 43.10 ± 10.49 years (range: 26-70 years). The majority of patients belonged to the 31-40 years age group, accounting for 20 (41.7%) patients, followed by the 41-50 years age group with 14 (29.2%) patients. Patients aged 51-60 years constituted seven (14.6%) cases, while four (8.3%) patients were in the 61-70-year age group. The least represented age group was 20-30 years, comprising three (6.3%) patients. There was a clear male predominance in the study population, with 34 (70.8%) males and 14 (29.2%) females. Regarding presenting complaints, left hip pain and bilateral hip pain were the most common symptoms, each reported by 17 (35.4%) patients, while right hip pain was noted in 14 (29.2%) patients. With respect to pain duration, the duration was not specified in 20 (41.7%) patients. Among patients with documented duration, pain lasting less than one year was observed in seven (14.6%) patients. A pain duration of one and two years was reported in five (10.4%) patients each, while four (8.3%) patients had pain for five years. A pain duration of three years was seen in three (6.3%) patients, whereas two (4.2%) patients each reported pain durations of four and six years (Table 1).

**Table 1:** Baseline demographic and clinical characteristics of patients undergoing total hip arthroplasty (THA) (n = 48). n: number of patients; THA: total hip arthroplasty.

Variable	Category	n (%)
Age group	20-30 years	3 (6.3)
	31-40 years	20 (41.7)
	41-50 years	14 (29.2)
	51-60 years	7 (14.6)
	61-70 years	4 (8.3)
Gender	Male	34 (70.8)
	Female	14 (29.2)
Chief complaint	Right hip pain	14 (29.2)
	Left hip pain	17 (35.4)
	Bilateral hip pain	17 (35.4)
Pain duration	<1 year	7 (14.6)
	1 year	5 (10.4)
	2 years	5 (10.4)
	3 years	3 (6.3)
	4 years	2 (4.2)
	5 years	4 (8.3)
	6 years	2 (4.2)
	Not specified	20 (41.7)

With respect to past surgical history, the majority of patients had no significant surgical history, accounting for 29 (60.4%) cases. Previous THA of the right hip was present in eight (16.7%) patients, while four (8.3%) patients had a history of hip core decompression. THA of the left hip was observed in five (10.4%) patients. Open reduction and internal fixation (ORIF) with plating for fracture of the left acetabulum and bilateral total knee replacement were each observed in one (2.1%) patient. Regarding pre-op diagnosis, AVN bilateral hip with right-sided predominance and AVN of the right hip were the most common indications for surgery, each accounting for 10 (20.8%) patients. AVN of the left hip with previous THA of the right hip was observed in eight (16.7%) patients. AVN bilateral hip with left-sided predominance and AVN of the left hip were each noted in seven (14.6%) patients, while AVN of the right hip with a previous THA of the left hip was present in five (10.4%) patients. Secondary osteoarthritis of the left hip with a post-operative left acetabulum fracture was observed in one (2.1%) patient. Regarding implant characteristics, uncemented implants were used in the majority of patients, accounting for 45 (93.8%) cases, whereas hybrid implants were used in three (6.3%) patients (Table 2).

**Table 2:** Past surgical history, preoperative diagnosis, and implant characteristics. THA: total hip arthroplasty; ORIF: open reduction and internal fixation; TKR: total knee replacement; AVN: avascular necrosis; OA: osteoarthritis.

Variable	Category	n (%)
Past surgical history	THA right hip	8 (16.7)
	Hip core decompression	4 (8.3)
	THA left hip	5 (10.4)
	ORIF with plating for fracture left acetabulum	1 (2.1)
	Bilateral TKR	1 (2.1)
	Nil significant	29 (60.4)
Preoperative diagnosis	AVN right hip	10 (20.8)
	AVN left hip	7 (14.6)
	AVN bilateral hip (right > left)	10 (20.8)
	AVN bilateral hip (left > right)	7 (14.6)
	AVN left hip with THA right hip	8 (16.7)
	AVN right hip with THA left hip	5 (10.4)
	Secondary OA left hip with post-operated left acetabulum fracture	1 (2.1)
Type of implant	Hybrid	3 (6.3)
	Uncemented	45 (93.8)

The baseline clinical and biochemical parameters of the study population were within acceptable ranges. The mean systolic blood pressure (SBP) was 113.75 ± 13.14 mmHg (95% CI: 109.94-117.56), while the mean diastolic blood pressure (DBP) was 72.46 ± 8.81 mmHg (95% CI: 69.91-75.02). The mean pulse rate (PR) was 87.54 ± 8.45 beats/min (95% CI: 85.09-89.99). The mean respiratory rate (RR) was 20.35 ± 2.16 breaths/min (95% CI: 19.72-20.98). Mean oxygen saturation (SpO₂) was 99.35 ± 1.13% (95% CI: 99.02-99.68). Among biochemical parameters, the mean hemoglobin (HB) level was 12.50 ± 2.27 g/dL (95% CI: 11.84-13.16). The mean prothrombin time (PT) was 13.97 ± 1.14 seconds (95% CI: 13.65-14.31), while the mean INR was 1.01 ± 0.23 (95% CI: 0.95-1.08). The mean total leukocyte count (TLC) was 8.66 ± 2.81 × 10³ cells/mm³ (95% CI: 7.85-9.48) (Table 3).

**Table 3:** Baseline clinical and biochemical parameters. SBP: systolic blood pressure; DBP: diastolic blood pressure; PR: pulse rate; RR: respiratory rate; SpO₂: peripheral oxygen saturation; HB: hemoglobin; PT: prothrombin time; INR: international normalized ratio; TLC: total leukocyte count; SD: standard deviation; CI: confidence interval.

Parameter	Mean ± SD	95% CI
SBP (mmHg)	113.75 ± 13.14	109.94-117.56
DBP (mmHg)	72.46 ± 8.81	69.91-75.02
PR (beats/min)	87.54 ± 8.45	85.09-89.99
RR (breaths/min)	20.35 ± 2.16	19.72-20.98
SpO₂ (%)	99.35 ± 1.13	99.02-99.68
HB (g/dL)	12.50 ± 2.27	11.84-13.16
PT (seconds)	13.97 ± 1.14	13.65-14.31
INR	1.01 ± 0.23	0.95-1.08
TLC (×10³ cells/mm³)	8.66 ± 2.81	7.85-9.48

Preoperatively, the majority of patients had ESR values within the 0-10 mm/hr range, accounting for 35 (72.9%) patients, while nine (18.8%) patients had ESR values between 11 and 20 mm/hr and four (8.3%) patients had values between 21 and 30 mm/hr. No patient demonstrated ESR values greater than 30 mm/hr during the pre-op period. On POD 3, a marked rise in ESR values was observed, with 41 (85.4%) patients demonstrating ESR levels greater than 30 mm/hr. ESR values between 21 and 30 mm/hr were observed in six (12.5%) patients, while only one (2.1%) patient had ESR values between 11 and 20 mm/hr. A similar distribution pattern persisted on POD 7, where 41 (85.4%) patients continued to demonstrate ESR values greater than 30 mm/hr. By POD 14, a gradual decline in ESR values was observed, with 27 (56.3%) patients showing ESR values greater than 30 mm/hr and 20 (41.7%) patients having values between 21 and 30 mm/hr. On POD 30, further reduction in ESR levels was noted, as 36 (75.0%) patients demonstrated ESR values between 21 and 30 mm/hr, while only nine (18.7%) patients continued to have ESR values greater than 30 mm/hr. The variation in ESR distribution across different postoperative time points was statistically significant (chi-square = 266.7, p < 0.0001) (Table 4).

**Table 4:** Distribution of erythrocyte sedimentation rate (ESR) status following THA. Statistical test used: chi-square test. Pre-op: preoperative; POD: postoperative day.

Time point	0-10 mm/hr n (%)	11-20 mm/hr n (%)	21-30 mm/hr n (%)	>30 mm/hr n (%)	Statistics
Pre-op	35 (72.9)	9 (18.8)	4 (8.3)	0 (0)	Chi-square: 266.7; p < 0.0001
POD 3	0 (0)	1 (2.1)	6 (12.5)	41 (85.4)	
POD 7	0 (0)	1 (2.1)	6 (12.5)	41 (85.4)	
POD 14	0 (0)	1 (2.1)	20 (41.7)	27 (56.3)	
POD 30	0 (0)	3 (6.3)	36 (75.0)	9 (18.7)	

The mean pre-op ESR level was 8.94 ± 6.58 mm/hr (95% CI: 7.03-10.85). Following surgery, ESR levels showed a marked rise on POD 3, reaching a mean value of 36.02 ± 5.90 mm/hr (95% CI: 34.31-37.74). Elevated ESR values persisted on POD 7 with a mean of 35.27 ± 6.16 mm/hr (95% CI: 33.48-37.06). Thereafter, a gradual decline in ESR levels was observed, with mean values decreasing to 32.19 ± 6.25 mm/hr (95% CI: 30.37-34.00) on POD 14 and 28.48 ± 5.61 mm/hr (95% CI: 26.85-30.11) on POD 30. However, ESR values remained higher than pre-op levels even at one month following surgery. Repeated measures ANOVA demonstrated a statistically significant change in ESR levels across the POD (F = 408.740, p < 0.001) (Table 5).

**Table 5:** Mean trend of ESR following THA. Statistical test used: repeated-measures analysis of variance (ANOVA). Pre-op: preoperative; POD: postoperative day; SD: standard deviation; CI: confidence interval.

Time point	Mean ± SD (mm/hr)	95% CI	Statistics
Pre-op	8.94 ± 6.58	7.03-10.85	F = 408.740; p < 0.001
POD 3	36.02 ± 5.90	34.31-37.74	
POD 7	35.27 ± 6.16	33.48-37.06	
POD 14	32.19 ± 6.25	30.37-34.00	
POD 30	28.48 ± 5.61	26.85-30.11	

Preoperatively, all 48 (100%) patients had CRP values within the 0-5 mg/L range. On POD 3, all patients demonstrated elevated CRP levels greater than 10 mg/L, and a similar pattern persisted on POD 7, where all 48 (100%) patients continued to have CRP values above 10 mg/L. By POD 14, 47 (97.9%) patients still had CRP values greater than 10 mg/L, while only one (2.1%) patient showed CRP normalization within the 0-5 mg/L range. On POD 30, a marked decline in CRP levels was observed, with 30 (62.5%) patients demonstrating CRP values within the normal range of 0-5 mg/L, 13 (27.1%) patients having mildly elevated values between 6 and 10 mg/L, and only five (10.4%) patients continuing to exhibit CRP values greater than 10 mg/L. The variation in CRP distribution across different postoperative time points was statistically significant (chi-square = 259.0, p < 0.0001) (Table 6).

**Table 6:** Distribution of C-reactive protein (CRP) status following THA.

Time point	0-5 mg/L n (%)	6-10 mg/L n (%)	>10 mg/L n (%)	Statistics
Pre-op	48 (100)	0 (0)	0 (0)	Chi-square: 259.0; p < 0.0001
POD 3	0 (0)	0 (0)	48 (100)	
POD 7	0 (0)	0 (0)	48 (100)	
POD 14	1 (2.1)	0 (0)	47 (97.9)	
POD 30	30 (62.5)	13 (27.1)	5 (10.4)	

The mean pre-op CRP level was 2.74 ± 1.43 mg/L (95% CI: 2.32-3.15). A marked pre-op rise in CRP levels was observed on POD 3, with a mean value of 131.19 ± 30.36 mg/L (95% CI: 122.37-140.00). Subsequently, CRP levels demonstrated a progressive decline, decreasing to 74.44 ± 22.93 mg/L (95% CI: 67.78-81.10) on POD 7 and 38.53 ± 18.39 mg/L (95% CI: 33.19-43.87) on POD 14. By POD 30, CRP levels had reduced considerably to 5.55 ± 3.70 mg/L (95% CI: 4.47-6.62), approaching pre-op values. Repeated measures ANOVA demonstrated a statistically significant change in CRP levels across the postoperative period (F = 499.360, p < 0.001) (Table 7).

**Table 7:** Mean trend of CRP following THA. Statistical test used: repeated-measures ANOVA. Pre-op: preoperative; POD: postoperative day; SD: standard deviation; CI: confidence interval.

Time point	Mean ± SD (mg/L)	95% CI	Statistics
Pre-op	2.74 ± 1.43	2.32-3.15	F = 499.360; p < 0.001
POD 3	131.19 ± 30.36	122.37-140.00	
POD 7	74.44 ± 22.93	67.78-81.10	
POD 14	38.53 ± 18.39	33.19-43.87	
POD 30	5.55 ± 3.70	4.47-6.62	

## Discussion

The present prospective observational study was conducted in the Department of Orthopaedics at Himalayan Institute of Medical Sciences, Dehradun, over a period of 12 months to evaluate postoperative trends of ESR and CRP following THA. A total of 48 patients were included, and serial measurements of ESR and CRP were obtained preoperatively and on PODs 3, 7, 14, and 30. The study aimed to establish the temporal pattern of these inflammatory markers following uncomplicated THA in the Indian population.

The mean age of patients in the present study was 43.10 ± 10.49 years, with most patients belonging to the 31-40-year age group. This age distribution was younger than that reported in most previous arthroplasty studies. Lang et al. reported mean ages of 66.32 ± 10.13 years and 62.28 ± 11.84 years in their study groups [10], while Zabair et al. observed a mean age of 55.95 ± 8.24 years [11]. Rohe et al. and Meier et al. also reported older patient populations with mean ages above 65 years [12, 13]. The relatively younger age group in the present study may be related to the higher prevalence of AVN in younger Indian patients undergoing THA. Male predominance was observed in the present study, with 70.8% males and 29.2% females. Similar male predominance was reported by Zabair et al. [11], whereas Aslani et al. observed female predominance in their cohort [14].

Regarding presenting complaints, left hip pain and bilateral hip pain were each observed in 35.4% of patients, while right hip pain was present in 29.2%. Rashid et al. reported relatively greater right-sided involvement in their series [15]. Pain duration varied considerably among patients, reflecting the chronic and progressive nature of hip pathology, similar to observations by Hamilton et al. [16].

Most patients (60.4%) had no significant prior surgical history, indicating that the majority underwent primary arthroplasty. Among patients with previous procedures, right THA, hip core decompression, and left THA were the most common surgeries. In contrast, Kahlenberg et al. reported implant loosening and polyethylene wear as major causes in revision arthroplasty cohorts [17]. The present study predominantly represented uncomplicated primary THA cases rather than revision arthroplasty.

The majority of patients had AVN-related indications for surgery. AVN of the right hip and bilateral AVN with right-sided predominance were each present in 20.8% of cases. Rohilla et al. similarly reported a high frequency of bilateral AVN involvement [18]. Uncemented implants were used in 93.8% of patients, whereas Garofalo et al. reported greater use of hybrid implants in their cohort [19]. The predominance of uncemented implants in the present study may reflect institutional preference and younger patient age.

Baseline clinical and biochemical parameters were within acceptable physiological limits. Mean systolic blood pressure, diastolic blood pressure, pulse rate, respiratory rate, and oxygen saturation indicated that the study population was clinically stable before surgery. Hemoglobin, PT, INR, and leukocyte counts were also within normal ranges. Hemoglobin levels are important while interpreting ESR because anemia may falsely elevate ESR independent of inflammation due to altered erythrocyte aggregation dynamics [20]. Similarly, leukocyte count reflects an immediate cellular inflammatory response but lacks the consistent postoperative trend observed with ESR and CRP [21]. Therefore, the relatively normal baseline hematological profile in the present study supports that postoperative changes in ESR and CRP were mainly attributable to surgical inflammation rather than underlying systemic abnormalities.

The present study demonstrated a characteristic postoperative ESR pattern following THA. The mean pre-op ESR was 8.94 ± 6.58 mm/hr, with most patients having values within the normal range. Following surgery, ESR increased markedly to 36.02 ± 5.90 mm/hr on POD 3 and remained elevated on POD 7. Thereafter, ESR gradually declined but remained above baseline even at POD 30. Distribution analysis showed that most patients had ESR values >30 mm/hr during the first postoperative week, followed by a shift toward the 21-30 mm/hr range by one month. These findings suggest that ESR normalizes slowly after THA and may remain elevated for several weeks despite uncomplicated recovery.

Similar postoperative ESR trends have been reported in previous studies. Zabair et al. observed a marked postoperative ESR rise from 27.3 ± 14.14 mm/hr preoperatively to 98.55 ± 31.02 mm/hr on POD 3, followed by gradual decline [11]. Sharma et al. and Kumar et al. also demonstrated early postoperative ESR peaks with delayed normalization [22, 23]. Aslani et al. similarly reported prolonged ESR elevation extending over several weeks [14]. Although the absolute ESR values in the present study were lower, the overall pattern of early rise followed by gradual decline was consistent with previous literature. The lower ESR levels observed in the present study may be related to younger patient age, lower baseline inflammatory burden, and exclusion of patients with chronic inflammatory conditions. These findings highlight that ESR should be interpreted according to postoperative trends rather than isolated values.

CRP demonstrated a more rapid and dynamic postoperative response compared with ESR. The mean pre-op CRP level was 2.74 ± 1.43 mg/L, and all patients had values within the normal range preoperatively. A marked postoperative increase was observed on POD 3, with mean CRP rising to 131.19 ± 30.36 mg/L. Subsequently, CRP declined progressively to 74.44 ± 22.93 mg/L on POD 7 and 38.53 ± 18.39 mg/L on POD 14. By POD 30, mean CRP had reduced to 5.55 ± 3.70 mg/L, approaching baseline levels. Most patients showed normalization or near-normalization of CRP by one month.

These findings are consistent with previous studies demonstrating early CRP peak and rapid decline after arthroplasty. Sharma et al. and Kumar et al. reported peak CRP levels around POD 3 with progressive reduction thereafter [22, 23]. Aslani et al. also observed near normalization of CRP within weeks following surgery [14]. Rohe et al. noted that CRP has limited diagnostic utility immediately after surgery because physiological postoperative elevation is expected [12]. However, Meier et al. emphasized that persistent or secondary elevation of CRP may suggest infection [13]. Thus, the present study supports the view that CRP is a sensitive marker of postoperative inflammation, but interpretation should be based on temporal trends rather than isolated early postoperative values.

The strengths of the present study include its prospective design and standardized serial monitoring of ESR and CRP at predefined postoperative intervals in a relatively homogeneous THA population. The findings provide a reference profile of the expected postoperative trends of these inflammatory markers following uncomplicated THA, which may assist clinicians in interpreting postoperative laboratory results. However, several limitations should be acknowledged. This was a single-center study with a relatively small, pragmatically determined sample size, which was not based on a formal repeated-measures power calculation and may limit the generalizability of the findings. Follow-up was limited to one month, and perioperative factors such as operative duration, blood loss, implant type, medications, and rehabilitation protocols were not specifically evaluated and may have influenced postoperative inflammatory marker levels. Furthermore, only uncomplicated THA cases were included; therefore, the present study was not designed to establish diagnostic thresholds or assess the diagnostic accuracy of ESR and CRP for detecting PJI. Future multicenter studies with larger sample sizes, longer follow-up, and inclusion of patients with postoperative complications are warranted to validate these findings and better define the clinical utility of postoperative inflammatory marker trends.

## Conclusions

In the present study, ESR and CRP demonstrated a consistent and predictable postoperative pattern following THA. Both markers showed distinct kinetics in the postoperative period. ESR exhibited a sharp increase with a relatively slower and prolonged decline, whereas CRP showed a rapid and pronounced early rise with a faster return toward baseline levels, reflecting their distinct biological kinetics. These findings highlight the differing temporal behavior of these inflammatory markers in the postoperative period. Based on these observations, the study delineates a reference postoperative trend pattern for ESR and CRP in patients undergoing THA in the studied population. These findings provide a reference pattern for the expected postoperative behavior of ESR and CRP following uncomplicated THA. This information may assist clinicians in interpreting postoperative inflammatory marker trends, although the present study was not designed to establish diagnostic thresholds or evaluate the ability of these biomarkers to diagnose PJI.
